# Supplementary material for: Exploring the effect of sequential antibiotic exposure in resistant Escherichia coli causing urinary tract infections: a proof of principle study
Source: Microbiol Spectr. 2025 Feb 4;13(3):e02525-24. doi: 10.1128/spectrum.02525-24 (PMC11878055; doi:10.1128/spectrum.02525-24)

**Supplementary appendix**

**Title:**

Exploring the effect of sequential antibiotic exposure in resistant *E. coli* causing urinary tract infections: A proof of principle study

**Authors:**

Lisa Göpel^1^, Laura Kirchhoff^1^, Olivia Gopleac^1^, Leif Tueffers^1^, Susanne Hauswaldt^1^, Sébastien Boutin^1,2,3^, Jan Rupp^1,2*^, Dennis Nurjadi^1,2*#^

**Affiliation:**

^1^ University of Lübeck and University Hospital Schleswig-Holstein Campus Lübeck, Institute of Medical Microbiology and Infectious Diseases Clinic, Lübeck, Germany

^2^ German Center for Infection Research (DZIF), Hamburg-Lübeck-Borstel-Riems, Lübeck, Germany

^3^ Airway Research Center North (ARCN), German Center for Lung Research (DZL), Lübeck, Germany

* contributed equally

**Table of contents**

[**Supplementary methods** 3](#_Toc185584502)

[**Dataset S1: Supplementary Data on Sequencing.** 7](#_Toc185584503)

[**Supplementary figure 1.** Area under the curve (AUC) analysis of sequential antibiotic exposure for *Escherichia coli* ATCC®25922 (A+B) and GM2163 (C+D). 8](#_Toc185584504)

[**Supplementary figure 2.** Growth curves of six clinical *E. coli* strains under sequential antibiotic treatment. 9](#_Toc185584505)

# **Supplementary methods**

*Strain collection*

Six *E. coli* strains isolated from urine samples of patients with urinary tract infections were selected for this study based on their susceptibility profile towards ciprofloxacin and/or mecillinam. The strains were randomly selected from the microbiological diagnostic laboratory from urine samples of patients with suspected UTIs (March 2023 to June 2023). The selection criteria were based on phenotypic resistance to either ciprofloxacin (minimum inhibitory concentration, MIC > 0.5 mg/L; Ecoli01, 02, and 03) or mecillinam (MIC > 8 mg/L; Ecoli04 and 05) or both (Ecoli0*6*) (Table 1). Species identification was performed using mass spectrometry (MALDI-TOF, Bruker Daltonics) and initial antibiotic susceptibility was performed using Vitek®2 (Biomerieux, Germany) and was interpreted according to the EUCAST clinical breakpoints (v13.1).

*Antibiotic susceptibility testing*

Antibiotic susceptibility testing (AST) for the *in vitro* experiments was conducted according to EUCAST recommendations. Microdilution for ciprofloxacin (Sigma-Aldrich, St. Louis, USA) and mecillinam (Sigma-Aldrich, St. Louis, USA) was performed using M9 medium supplemented with glucose (2 g/L), citrate (0.5 g/L) and casamino acids (1 g/L). A 96-well microplate was prepared with 2-fold dilutions of ciprofloxacin and mecillinam, with concentrations ranging from 0.008 to 128 µg/mL. To achieve a final inoculum concentration of 5 x 10^5^ CFU/mL in the microplate, a 0.5 McFarland standard (comparable to a bacterial suspension of 1.5 x 10^8^ CFU/mL) was prepared in M9 medium and diluted 1:100 in fresh M9 medium. The microplate was incubated at 37 °C for 18 hours, after which the MIC was determined as the lowest concentration of antimicrobial agent that inhibited visible growth of the bacteria.

*Growth measurement*

Bacteria (1.5 x 10^6^ CFU/mL) were pretreated with either ciprofloxacin or mecillinam (0.75 x MIC) and incubated for one hour at 37°C on a shaking incubator at 110 rpm. Subsequently, the liquid cultures were centrifuged at 3220 x g for five minutes, the supernatant was discarded, and the bacterial pellets were resuspended in fresh M9 medium. The cells were then exposed to the second antibiotic (either ciprofloxacin or mecillinam, depending on the pretreatment) at four different concentrations (0.0375 x MIC; 0.075 x MIC; 0.375 x MIC and 0.75 x MIC) in a microtitre plate, with each well containing 100 µl. The plate was incubated at 37°C with constant shaking. Optical density was measured automatically at 600 nm every 15 minutes for eight or 12 hours using an Epoch 2NS plate reader (Agilent Technologies, Santa Clara, USA). All tests were performed in triplicate (biological and technical).

*Intact cell counting*

The impedance flow cytometer BactoBox (SBT Instruments, Herlev, Denmark) was used to perform intact cell counting. For each liquid culture during exposure to the second antibiotic (at timepoints 0h, 4h, and 8h), serial dilutions were performed in 15 mL Falcons containing a volume of 10 mL 1/10 Dulbecco´s phosphate-buffered saline buffer (Life Technologies, Carlsbad USA) until the final concentration was within the detection range (10^4^-10^6^ total cells/mL). Tests were performed in biological triplicate.

*Checkerboard assay to test for synergy*

A modified checkerboard assay was used to determine the synergistic effect of ciprofloxacin and mecillinam (1). Briefly, a 96-well microplate containing concentrations of ciprofloxacin and mecillinam based on the respective MICs of the strains was prepared with a fresh M9 medium. For a final inoculum concentration in the microplate of 5 x 10^5^ CFU/mL, 0.5 McFarland standard was adjusted in M9 medium and diluted 1:100 in M9 medium. The microplate was incubated at 37°C and the optical density at 600 nm was measured after 18±2 h using an Infinite M200 PRO (Tecan, Switzerland).

The following formula was used to calculate the fractional inhibitory concentration index (FICI)= (MIC_substance_ _A_ in combination/MIC_substance A_ alone) + (MIC_substance B_ in combination/MIC_substance B_ alone). Effects of the antibiotic agent combinations were classified according to the following criteria: (1) FICI ≤ 0.5, synergistic effects; (2) 0.5 < FICI < 4, no interactions; (3) FICI ≥ 4.0, antagonistic effects.

*Whole genome sequencing*

Genomic DNA was extracted from an overnight culture on Columbia blood agar using the DNeasy Blood and Tissue minikit (Qiagen, GmbH) following the manufacturer’s instructions. Library preparations were performed using the DNA Prep Kit (Illumina) and sequenced using the NextSeq 2000 instrument (2 x 100 cycles).

Raw fastq files were trimmed for adapters and low-quality reads using fastp (v0·23·1 with parameters -q = 30 and -l = 45) (2) and assembled with SPAdes 3.15.5 (with the option —careful and—only-assembler) (3). A curation of the draft genomes was performed by removing contigs with a length <500 bp and/or coverage < 10× and the quality of the assembly was assessed using Quast (v5·0·2) (4). The species identification of each draft genome was done using mash (sub-command screen) by screening each draft genome to a database composed of a representative genome of each species present in the Microbial Genomes resource (<https://www.ncbi.nlm.nih.gov/genome/microbes/>). The complete draft genomes were processed through available databases using Abricate (<https://github.com/tseemann/abricate>) to identify antimicrobial resistance (NCBI, CARD, ARG-ANNOT, ResFinder, MEGARES databases) (5, 6). Phylogroups of *E. coli* genomes were determined by using the web-based tool ClermontTyper (7) and serotypes were determined by applying the web-based SerotypeFinder 2.0, provided by the Center for Genomic Epidemiology (8). Genomes were annotated using Prokka v.1.14.5 (9) and a gene presence/absence matrix was built using Roary v.3.13.0 (10).

*Statistical analysis and data visualization*

Data visualization and statistical analysis were performed using R 4.3.3 with packages Growthcurver and ggplot2.

*Ethical considerations*

Admission to use clinical bacterial strains without patient data was obtained by a waiver of consent.

**References**

1. Bellio P, Fagnani L, Nazzicone L, Celenza G. 2021. New and simplified method for drug combination studies by checkerboard assay. MethodsX 8:101543.

2. Chen S, Zhou Y, Chen Y, Gu J. 2018. fastp: an ultra-fast all-in-one FASTQ preprocessor. Bioinformatics 34:i884-i890.

3. Bankevich A, Nurk S, Antipov D, Gurevich AA, Dvorkin M, Kulikov AS, Lesin VM, Nikolenko SI, Pham S, Prjibelski AD, Pyshkin AV, Sirotkin AV, Vyahhi N, Tesler G, Alekseyev MA, Pevzner PA. 2012. SPAdes: a new genome assembly algorithm and its applications to single-cell sequencing. J Comput Biol 19:455-77.

4. Gurevich A, Saveliev V, Vyahhi N, Tesler G. 2013. QUAST: quality assessment tool for genome assemblies. Bioinformatics 29:1072-5.

5. Feldgarden M, Brover V, Haft DH, Prasad AB, Slotta DJ, Tolstoy I, Tyson GH, Zhao S, Hsu CH, McDermott PF, Tadesse DA, Morales C, Simmons M, Tillman G, Wasilenko J, Folster JP, Klimke W. 2019. Validating the AMRFinder Tool and Resistance Gene Database by Using Antimicrobial Resistance Genotype-Phenotype Correlations in a Collection of Isolates. Antimicrob Agents Chemother 63.

6. Carattoli A, Zankari E, Garcia-Fernandez A, Voldby Larsen M, Lund O, Villa L, Moller Aarestrup F, Hasman H. 2014. In silico detection and typing of plasmids using PlasmidFinder and plasmid multilocus sequence typing. Antimicrob Agents Chemother 58:3895-903.

7. Beghain J, Bridier-Nahmias A, Le Nagard H, Denamur E, Clermont O. 2018. ClermonTyping: an easy-to-use and accurate in silico method for Escherichia genus strain phylotyping. Microb Genom 4.

8. Joensen KG, Tetzschner AM, Iguchi A, Aarestrup FM, Scheutz F. 2015. Rapid and Easy In Silico Serotyping of Escherichia coli Isolates by Use of Whole-Genome Sequencing Data. J Clin Microbiol 53:2410-26.

9. Seemann T. 2014. Prokka: rapid prokaryotic genome annotation. Bioinformatics 30:2068-9.

10. Page AJ, Cummins CA, Hunt M, Wong VK, Reuter S, Holden MT, Fookes M, Falush D, Keane JA, Parkhill J. 2015. Roary: rapid large-scale prokaryote pan genome analysis. Bioinformatics 31:3691-3.

**Dataset S1: Supplementary Data on Sequencing.** Sequencing data, including antibiotic resistance genes, virulence determinants, and plasmid Inc-types are provided in a separate excel file.

**Supplementary figure 1.** Area under the curve (AUC) analysis of sequential antibiotic exposure for *Escherichia coli* ATCC®25922 (A+B) and GM2163 (C+D). Two distinct concentrations of pre-treatment were employed, namely 0.075 x MIC (A+C) and 0.75 x MIC (B+D). The main-treatment concentrations used for ATCC®25922 were 0.375 x MIC for fosfomycin (FOS), mecillinam (MEC), and trimethoprim-sulfamethoxazole (SXT) and 0.75 x MIC for ciprofloxacin (CIP). The main-treatment concentrations employed for GM2163 differed only in the case of FOS, which was set at 0.75 x MIC, in comparison to the concentrations used for ATCC®25922. The blue arrows indicate a reduction in bacterial growth following sequential exposure compared to single exposure, while the red arrows demonstrate an enhanced bacterial growth following sequential exposure.


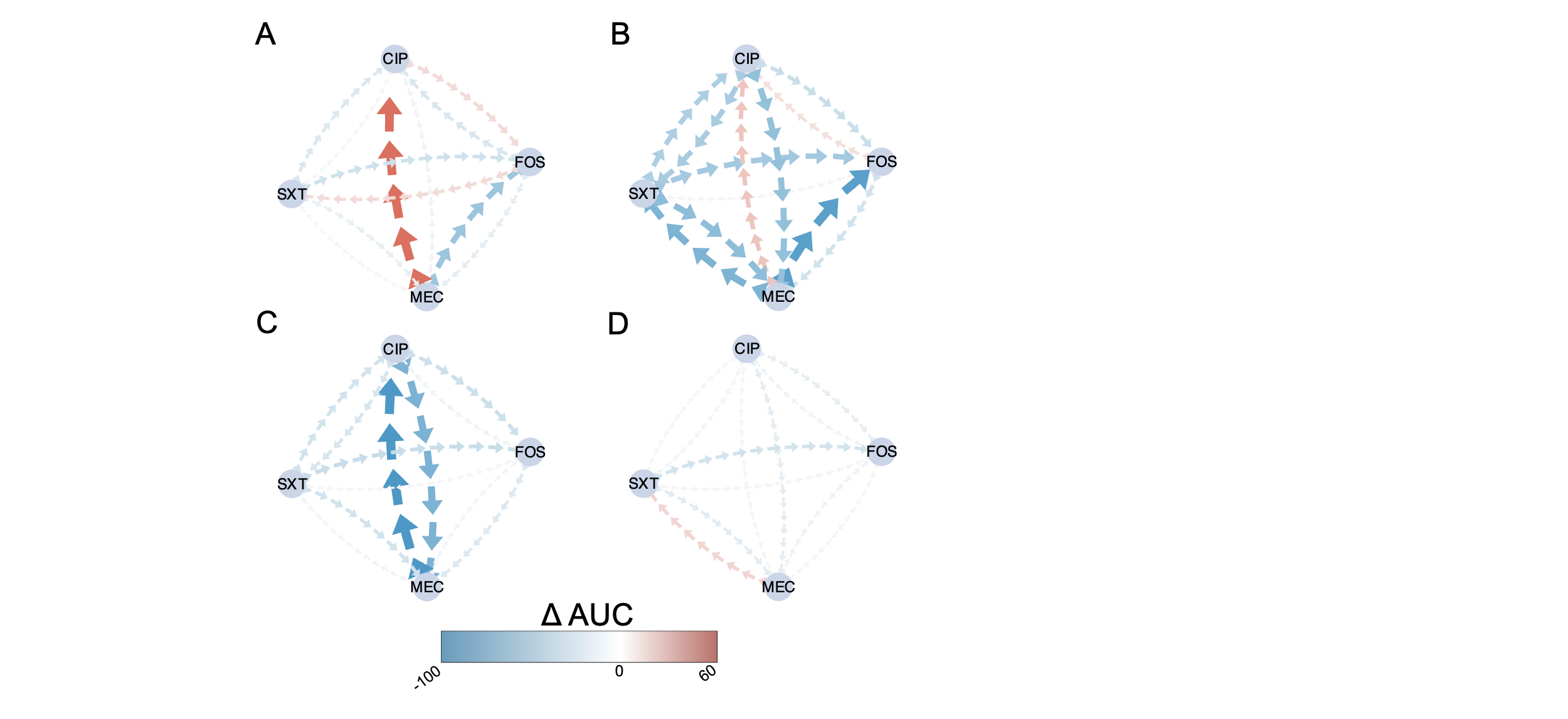


**Supplementary figure 2.** Growth curves of six clinical *E. coli* strains under sequential antibiotic treatment. **(a)** Isolates were incubated with mecillinam (0.75 x MIC) for one hour, followed by ciprofloxacin treatment (0.0375 x MIC; 0.075 x MIC; 0.375 x MIC; 0.75 x MIC) for 12 hours. The dashed lines represent the result of mecillinam pre-treatment alone (in black) and mecillinam pre-treatment followed by ciprofloxacin exposure of varying concentrations (blue). The full blue lines represent the various concentrations of ciprofloxacin employed as a standalone treatment. A reduction in the growth of Ecoli04 – 06 was observed during the first six to ten hours when sequential antibiotic exposure was employed, in comparison to single exposure. Following the initial decrease in growth, there was either no difference (Ecoli05) or even an increase in bacterial growth (Ecoli04 and 06) with sequential exposure compared to single exposure with ciprofloxacin for 12 hours. **(b)** Isolates were incubated with ciprofloxacin (0.75 x MIC) for one hour, followed by mecillinam exposure (0.0375 x MIC; 0.075 x MIC; 0.375 x MIC; 0.75 x MIC) for eight hours. The dashed lines represent the result of ciprofloxacin pre-treatment alone (in black) and ciprofloxacin pre-treatment followed by mecillinam exposure of varying concentrations (red). The full red lines represent the various concentrations of mecillinam employed as a standalone treatment.


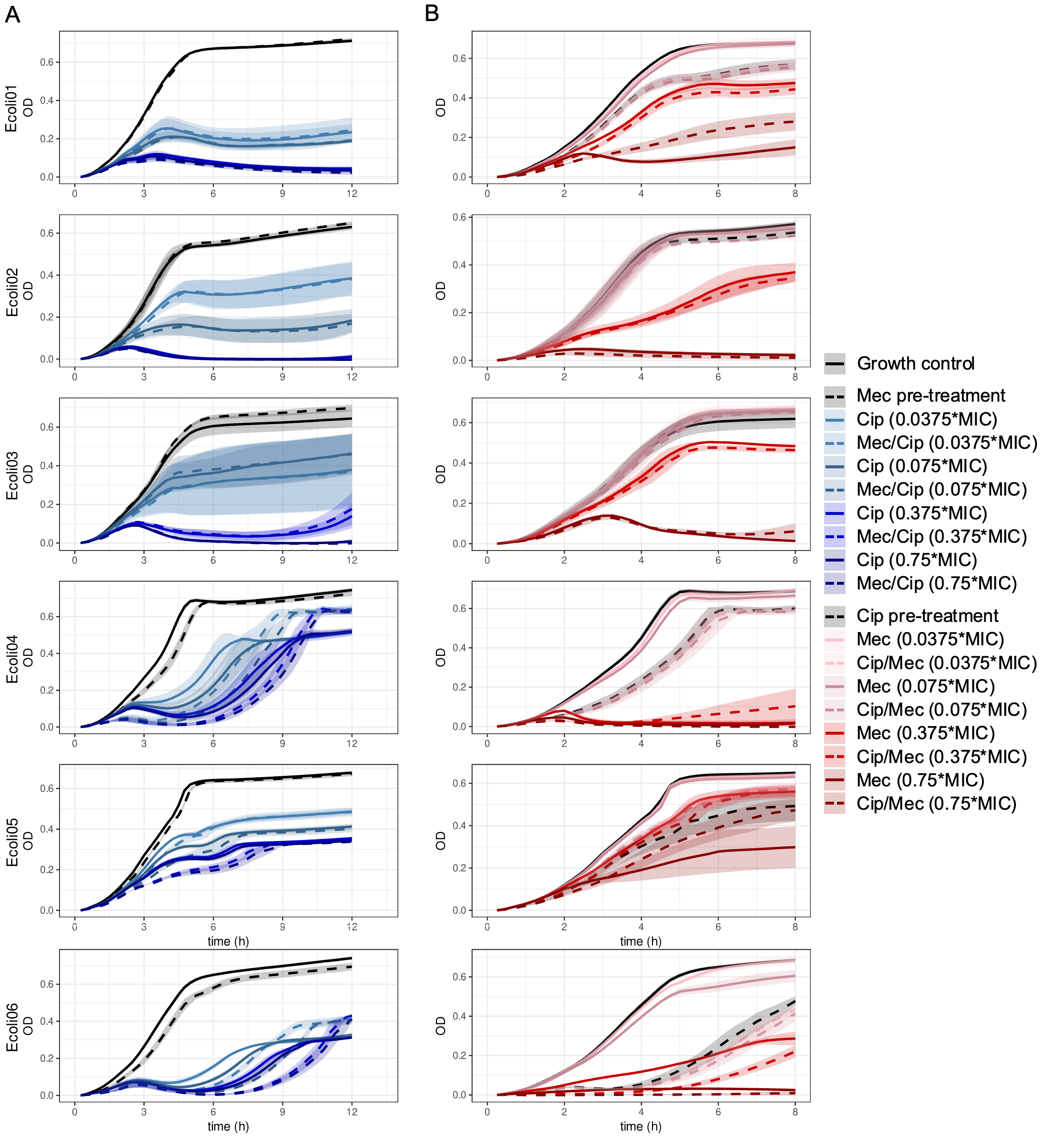

Supplement: Supplemental material — Supplemental methods; Figures S1 and S2. [file spectrum.02525-24-s0002.docx]
